# Author Correction: Rift-induced disruption of cratonic keels drives kimberlite volcanism

**DOI:** 10.1038/s41586-023-06960-2

**Published:** 2023-12-18

**Authors:** Thomas M. Gernon, Stephen M. Jones, Sascha Brune, Thea K. Hincks, Martin R. Palmer, John C. Schumacher, Rebecca M. Primiceri, Matthew Field, William L. Griffin, Suzanne Y. O’Reilly, Derek Keir, Christopher J. Spencer, Andrew S. Merdith, Anne Glerum

**Affiliations:** 1 School of Ocean and Earth Science, University of Southampton, Southampton, UK; 2 School of Geography, Earth and Environmental Sciences, University of Birmingham, Birmingham, UK; 3 Helmholtz Centre Potsdam – GFZ German Research Centre for Geosciences, Potsdam, Germany; 4 University of Potsdam, Potsdam-Golm, Germany; 5 Department of Geology, Portland State University, Portland, OR USA; 6 Mayfield, Wookey Hole, Wells, UK; 7 GEMOC ARC National Key Centre, Department of Earth and Environmental Sciences, Macquarie University, Sydney, New South Wales Australia; 8 Dipartimento di Scienze della Terra, Universita degli Studi di Firenze, Florence, Italy; 9 Department of Geological Sciences and Geological Engineering, Queen’s University, Kingston, Ontario Canada; 10 School of Earth and Environment, University of Leeds, Leeds, UK

**Keywords:** Volcanology, Tectonics, Geochemistry, Geodynamics

Correction to: *Nature* 10.1038/s41586-023-06193-3 Published online 26 July 2023

This article was originally published under a standard Springer Nature licence (© The Author(s), under exclusive licence to Springer Nature Limited). It is now available as an open-access paper under a Creative Commons Attribution 4.0 International licence, © The Author(s). The status has been updated in the HTML and PDF versions of the article.

