# Supplementary figures and images for: Rift-induced disruption of cratonic keels drives kimberlite volcanism
Source: Nature. 2023 Jul 26;620(7973):344–50. doi: 10.1038/s41586-023-06193-3 (PMC10727985; doi:10.1038/s41586-023-06193-3)

dFrag (9 Myr) leading Kimberlite count; 500–0Ma (5Myr res)

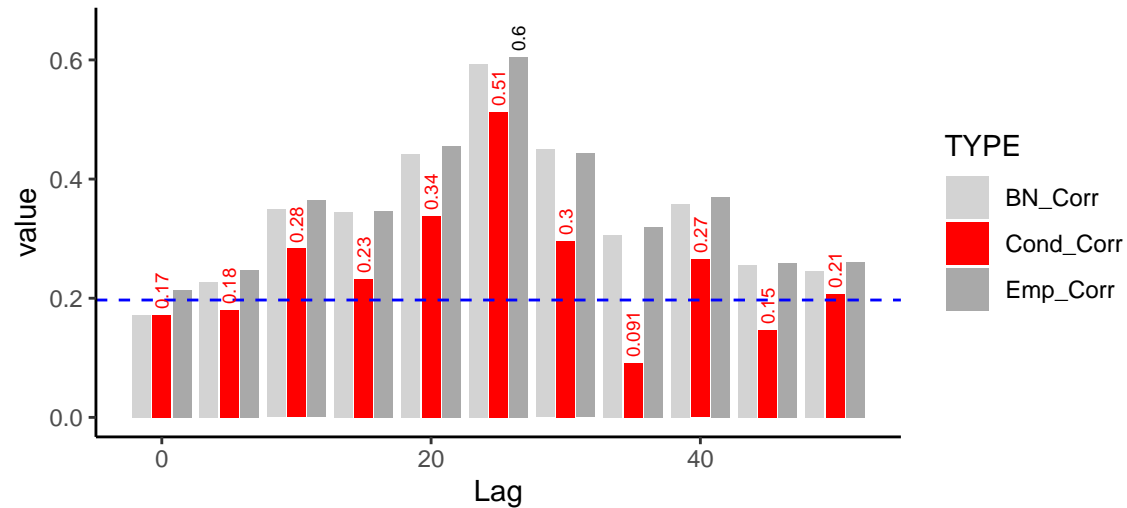

Supplement: Supplementary file 9 — Source Data Extended Data Fig. 2 [file 41586_2023_6193_MOESM9_ESM.zip › 41586_2023_6193_MOESM9_ESM/CORRELATIONS_500Myr_5MryRes_dFrag9Myr_Kim.pdf]

# dFrag (9 Myr) leading Kimberlite count; 500–0Ma (5Myr)

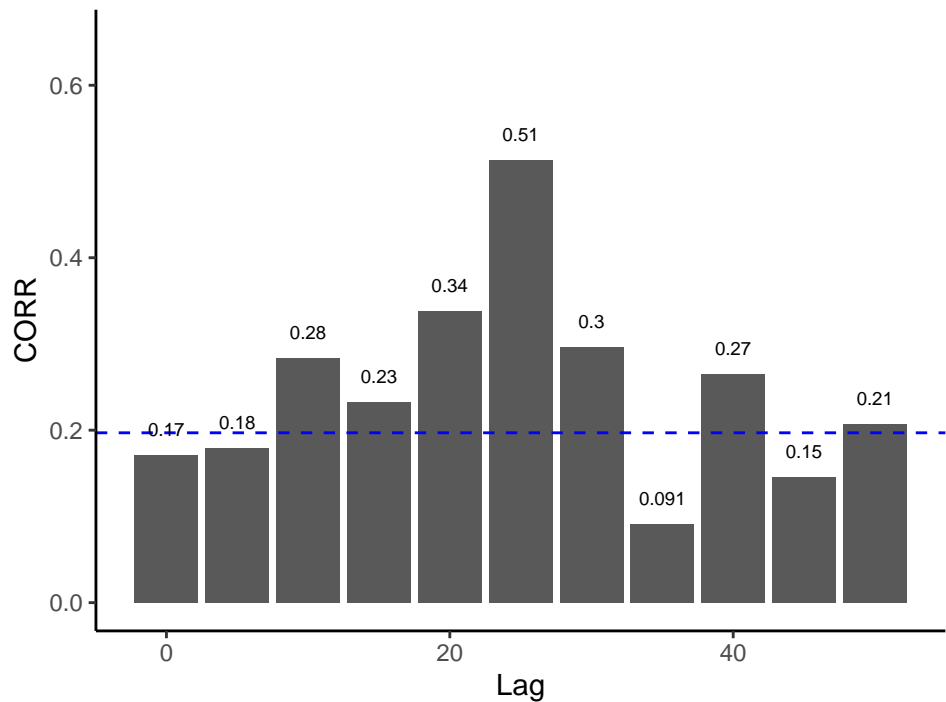

Supplement: Supplementary file 9 — Source Data Extended Data Fig. 2 [file 41586_2023_6193_MOESM9_ESM.zip › 41586_2023_6193_MOESM9_ESM/COND_CORR_500Myr_5MryRes_dFrag9Myr_Kim.pdf]

LIP start leading dFrag (9 Myr); 1000–0Ma (5Myr res)

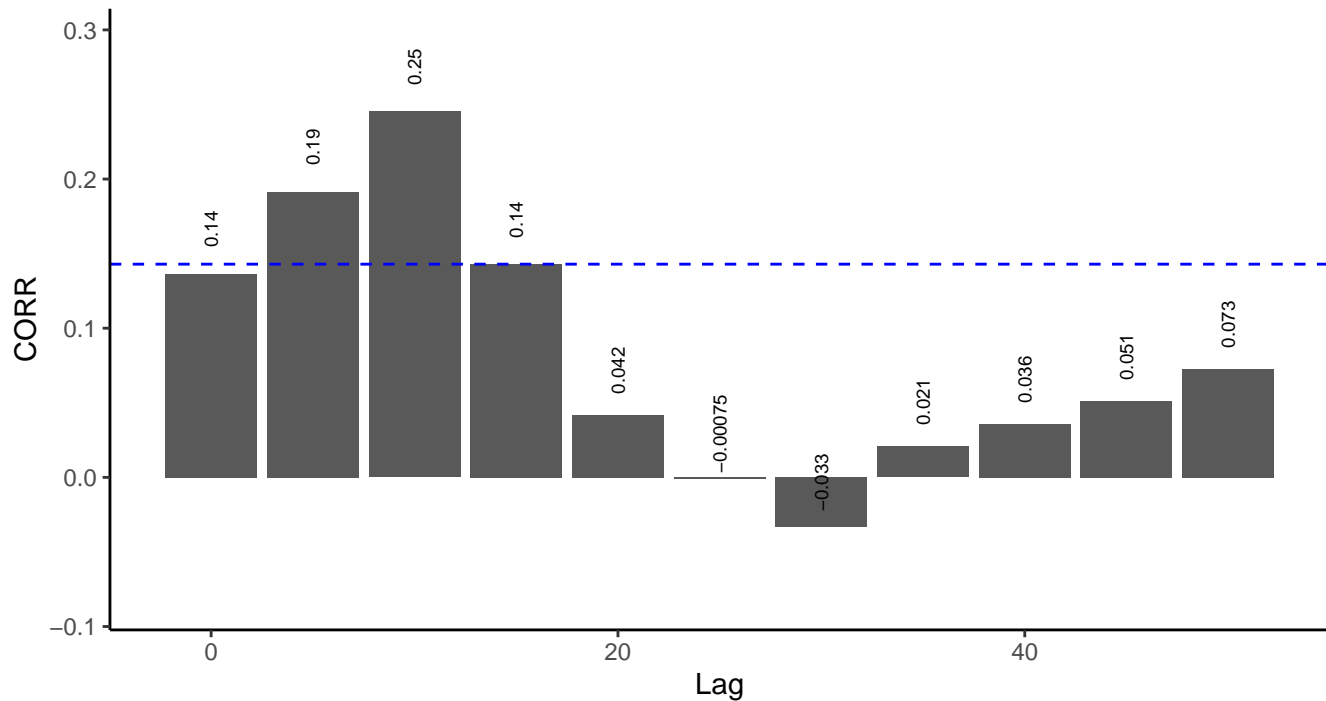

Supplement: Supplementary file 13 — Source Data Extended Data Fig. 6 [file 41586_2023_6193_MOESM13_ESM.zip › UNINET_1000Myr_5MryRes_LIPstartSum5Myr_dFrag9Myr/COND_CORR_1000Myr_5MryRes_LIPstartSum5Myr_dFrag9Myr.pdf]
